
JOURNAL INFORMATION
==============================
NLM Title Abbreviation: Biospektrum (Heidelb)
Journal ID: Biospektrum (Heidelb)
NLM Journal ID: 9615685

Biospektrum

ISSN: 0947-0867
EISSN: 1868-6249

ARTICLE INFORMATION
==============================
PMCID: PMC9178215
PMID: 35698575
DOI: 10.1007/s12268-022-1772-z
Article ID: 1772
Article version: 1
Subjects: Wissenschaft · Methoden

Hochaufgelöste Mikrobenporträts mit hoher Tiefenschärfe

https://orcid.org/0000-0002-0161-8326 Schmidt Matthias matthias.schmidt@ufz.de https://www.ufz.de/provis

grid.7492.8 0000 0004 0492 3830 ProVIS - Zentrum für Chemische Mikroskopie Abteilung Isotopen Biogeochemie Helmholtz-Zentrum für Umweltforschung GmbH - UFZ, Permoserstraße 15, D-04318 Leipzig, Deutschland
Electronic publication date: 2022 Jun 9
Print publication date: 2022
Volume: 28
Issue: 4
First page: 377
Last page: 380
Copyright: © Der Autor 2022
License: Open Access: Dieser Artikel wird unter der Creative Commons Namensnennung 4.0 International Lizenz veröffentlicht, welche die Nutzung, Vervielfältigung, Bearbeitung, Verbreitung und Wiedergabe in jeglichem Medium und Format erlaubt, sofern Sie den/die ursprünglichen Autor(en) und die Quelle ordnungsgemäß nennen, einen Link zur Creative Commons Lizenz beifügen und angeben, ob Änderungen vorgenommen wurden. Die in diesem Artikel enthaltenen Bilder und sonstiges Drittmaterial unterliegen ebenfalls der genannten Creative Commons Lizenz, sofern sich aus der Abbildungslegende nichts anderes ergibt. Sofern das betreffende Material nicht unter der genannten Creative Commons Lizenz steht und die betreffende Handlung nicht nach gesetzlichen Vorschriften erlaubt ist, ist für die oben aufgeführten Weiterverwendungen des Materials die Einwilligung des jeweiligen Rechteinhabers einzuholen. Weitere Details zur Lizenz entnehmen Sie bitte der Lizenzinformation auf http://creativecommons.org/licenses/by/4.0/deed.de.
License URL: https://creativecommons.org/licenses/by/4.0/

issue-copyright-statement: © The Author(s), under exclusive licence to Springer-Verlag GmbH Deutschland, ein Teil von Springer Nature 2022

==============================
The past decade has seen the advent of a new imaging technique in the life sciences that is ideal for microbiological applications: the helium ion microscope (HIM). Its lateral resolution is better than one nanometre, it has a large depth-of-field and can image non-conductive specimens which renders the tool ideal for studying microbiological objects such as microbes attached to surfaces, microbial biofilms and viruses. Here we compare HIM with electron microscopy techniques and highlight selected examples that demonstrate the new possibilities for microbiology opened up by this technique.

■ Bereits wenige Jahre nach der Erfindung des Transmissionselektronenmikroskops (TEM) wurde sein enormes Potenzial für die Mikrobiologie [1, 2] und Virologie [3] erkannt. Mittels TEM entstand in der Folge ein umfassendes Bild des Aufbaus mikrobieller Zellen sowie der Ultrastruktur ihrer Zellbestandteile, das sich bis heute durch immer ausgefeiltere Präparationstechniken und verbesserte Mikroskope verfeinert.

Ein Nachteil der TEM besteht in der Notwendigkeit ultradünner Proben von weniger als 100 Nanometer Dicke, was normalerweise durch Einbetten der Mikroben in Kunstharz und anschießender Ultramikrotomie erreicht wird. Während dies für planktonische Zellkulturen kein Problem darstellt, gestaltet sich die Untersuchung mikrobieller Zellen auf harten Oberflächen deutlich schwieriger, da harte Objekte nicht mit Mikrotommessern geschnitten werden können. Beginnend mit der Abbildung von Bakterien in Bodenproben [4] wird deshalb seit den 1960er-Jahren die zum TEM komplementäre Rasterelektronenmikroskopie (REM) häufig zur Mikroskopie von Mikroben verwendet.

Die Probenpräparation für REM ist vergleichsweise einfach: Einbetten und Schneiden der Probe sind nicht nötig, jedoch muss die Oberfläche durch Aufbringen einer nur wenige Nanometer dicken Metall- bzw. Kohleschicht elektrisch leitfähig gemacht werden, um eine – die Bildqualität stark beeinträchtigende – elektrische Aufladung zu verhindern. Durch die einfache Präparation der Proben sowie die plastisch wirkenden, pseudo-dreidimensionalen Aufnahmen, wurde die REM zu einem Standardwerkzeug der mikroskopischen Bildgebung von Mikroorganismen und in geringerem Maße auch Viren, obgleich ihre laterale Auflösung geringer als die der TEM ist.

## Hohe Auflösung, Tiefenschärfe und Ladungskompensation

Seit etwa 15 Jahren existiert mit der Heliumionenmikroskopie (HIM) eine weitere hochauflösende mikroskopische Technik [5], die der REM in vielen Aspekten gleicht, jedoch einige entscheidende Vorteile für die mikrobiologische Bildgebung hat. Über die in der evakuierten Analysekammer befindliche Probe wird bei der HIM anstelle eines Elektronenstrahls ein fokussierter Strahl von Heliumionen gerastert, der dort, analog zum REM, Sekundärelektronen erzeugt. Wegen des großen Massenunterschieds der Stoßpartner – ein Heliumion ist rund 8.000-mal schwerer als ein Elektron – sind diese jedoch deutlich niederenergetischer als im REM. Daher gelingt es nur den in einer ultradünnen Schicht wenige Nanometer unter der Oberfläche gebildeten Sekundärelektronen,

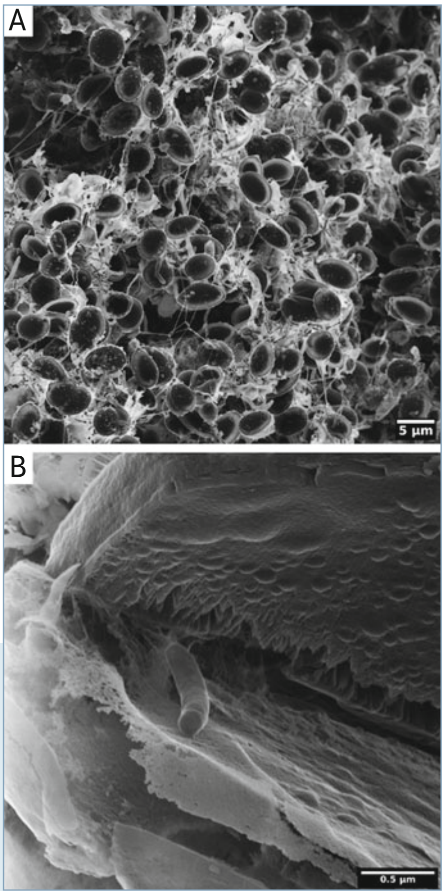

► **Abb. 1**: **A**, Heliumionenmikrografie eines von *Chlorella*-Mikroalgen gebildeten Biofilms, der durch die große Tiefenschärfe trotz starker Topografie nahezu überall scharf fokussiert ist. Die hohe Oberflächensensitivität des HIM erlaubt eine kontrastreiche Abbildung der exozellulären Polymersubstanzen. Quelle: Moreno-Osorio *et al.* [7, 8], CC BY 4.0. **B**, Heliumionenmikrografie eines nitratreduzierenden $Fe(II)$-oxidierenden Bakteriums in einem Eisenmineral. Die Ladungskompensation das HIM erlaubte es, Bakterium und Mineral ohne Metallisierung der Oberfläche abzubilden, sodass die Oberfläche als nativ angesehen werden kann. Bisher unveröffentlichte Aufnahme aus der Arbeit von Byrne *et al.* [9].

die Probe zu verlassen und somit zur Bildgebung beizutragen. Dies hat zur Folge, dass das HIM nicht nur eine hohe laterale Auflösung von etwa 0,5 Nanometer erreicht, sondern zugleich eine dem REM überlegene Oberflächensensitivität hat. Die hohe Masse der Heliumionen und ihre niedrige Geschwindigkeit (verglichen mit Elektronen gleicher Energie) machen die Technik außerdem recht unempfindlich gegenüber magnetischen Störfeldern und verleihen der HIM eine ungefähr fünfmal höhere Tiefenschärfe verglichen mit der REM. Ein weiterer Vorteil des HIM ist die Möglichkeit der Ladungskompensation. Dazu werden nach jeder gerasterten Zeile Ionenstrahl und Elektronendetektor abgestellt und die in Probe eingebrachten positiven Ladungen mittels einer „Elektronendusche“ aus einer Elektronenkanone kompensiert.

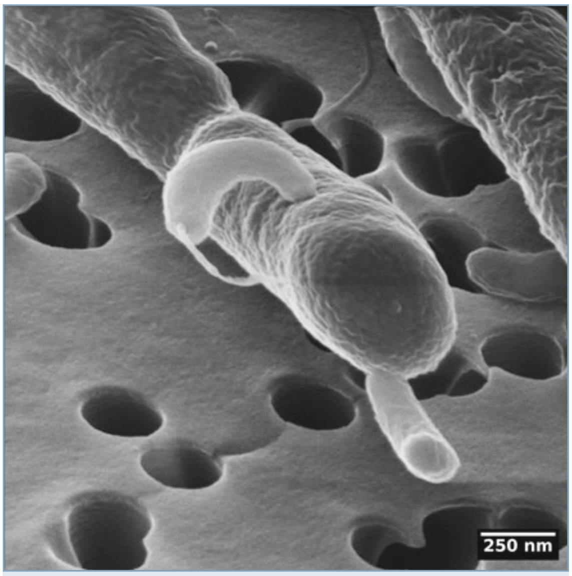

▲ **Abb. 2:** Die Infektion eines *Escherichia coli*-Bakteriums durch das räuberische Bakterium *Bdellovibrio bacteriovorus*, eingefangen durch Heliumionenmikrografie. Probe: Nedal Said und Antonis Chatzinotas, Helmholtz-Zentrum für Umweltforschung GmbH – UFZ, Leipzig. Abbildung aus [11], reproduziert mit Einwilligung des Wiley Verlags, © Wiley.

## Anforderungen an das Präparat

Wie für jede Mikroskopie liegt der Schlüssel zum gelungenen Bild auch bei der HIM in der richtigen Probenpräparation. Generell gilt, dass die Probe vakuumkompatibel, also fixiert und frei von Wasser bzw. im Vakuum der Analysekammer verdampfenden Substanzen sein muss. Jede für REM präparierte Probe kann im Prinzip auch mittels HIM untersucht werden, wobei letztere jedoch dank Ladungskompensation keine elektrisch leitfähige Oberfläche erfordert. Protokolle zur Präparation biologischer Proben für die HIM bestehen typischerweise aus den Schritten chemische Fixierung (z. B. mittels Formalin oder Glutaraldehyd), ggf. Nachfixierung durch ein starkes Oxidationsmittel (z. B. Osmiumtetroxid oder Kaliumpermanganat) sowie langsame Entwässerung in einer ansteigenden Ethanolreihe und Trocknung (z. B. Kritischpunkttrocknung).

## Einsatz für Umweltmikrobiologie, mikrobielle Biofilme

Hohe Auflösung, Tiefenschärfe und Ladungskompensation prädestinieren die HIM geradezu für die Untersuchung umweltmikrobieller Proben und mikrobieller Biofilme, da deren Oberflächen oft von einer starken Topografie geprägt und elektrisch isolierend sind. Zur Illustration dessen zeigt **Abbildung 1** exemplarisch HIM-Aufnahmen eines von *Chlorella*-Mikroalgen gebildeten Biofilms (**Abb. 1A**), sowie eines mikroaerophilen Fe(II)-oxidierenden Bakteriums auf einem Mineral (**Abb. 1B**).

Auf der Aufnahme des Biofilms (**Abb. 1A**) treten die exozellulären Polymersubstanzen zwischen den Algenzellen durch die hohe Oberflächensensitivität der HIM deutlich hervor und die Oberflächenstrukturen der Zellen sowie vereinzelte Bakterien darauf sind gut erkennbar. Das tiefe Eindringen der Heliumionen in die Zellen bewirkt, dass auch in ihren Rückseiten Sekundärelektronen erzeugt werden, wodurch die Zellen transluzent wirken.

Es verwundert nicht, dass die ersten HIM-Untersuchungen an Bakterien keine Modellkulturen zum Gegenstand hatten, sondern nitratreduzierende, Fe(II) zu Fe(III) oxidierende Bakterien auf Eisenmineralien [6]. Die harten Mineralphasen der Proben ließen keine Ultramikrotomieschnitte für TEM-Untersuchungen zu. REM wäre natürlich möglich, erfordert aber eine Metallisierung der Probenoberfläche zur Verhinderung elektrischer Aufladung. Die Mineral- und Mikrobenoberflächen würden durch die Metallschicht jedoch verändert und durch die starke Probentopografie könnte es zu Abschattungseffekten kommen, was in der Folge zu falscher Bildinterpretation führen könnte. Im Gegensatz dazu machen seine große Tiefenschärfe und die Ladungskompensation das HIM zum idealen Mikroskop zur Untersuchung solcher Proben, wie **Abbildung 1B** demonstriert.

## Bakteriophagen und bakterielle Prädatoren

Die hohe Auflösung der HIM legt ihre Verwendung zur Untersuchung von Virenpartikeln nahe. Die erste Arbeit dazu publizierten Miika Leppänen *et al.* 2017 und zeigten anhand eindrucksvoller Aufnahmen der Infektion von *Escherichia coli* mit dem T4-Phagen das große Potenzial der HIM für die Phagenforschung und -therapie auf [10].

In einer ähnlichen Arbeit berichteten Nedal Said *et al.* über ihre Studien zum Lebenszyklus des prädatorischen Bakteriums *Bdellovibrio bacteriovorus*, das Gram-negative Beutebakterien infiziert, penetriert und sich in ihnen vermehrt [11]. Dank der hohen Oberflächensensitivität konnte mittels HIM das Anhaften des Räubers an der Beutezelle sowie die darauffolgende Entwicklung von Bdelloplasten, deren Lyse und Freisetzung neuer Bdellovibrien detailliert dargestellt werden. **Abbildung 2** zeigt die HIM-Aufnahme einer Infektion eines *E. coli* durch *B. bacteriovorus*, 20 Minuten nachdem die Kulturen in Kontakt gebracht wurden.

## SARS-CoV-2

In der aktuellen Pandemie leistet die HIM einen wertvollen Beitrag zum Verständnis der Infektion mit SARS-CoV-2. Natalie Frese *et al.* zeigten mittels HIM, wie mit SARS-CoV-2 infizierte Vero-E6-Zellen neue Virenpartikel durch Knospung ihrer Zellmembran freisetzen [12]. Die hohe Auflösung ermöglicht es hier, zwischen Virenknospen und auf der Membran liegenden Virenpartikeln zu unterscheiden. **Abbildung 3** zeigt eine eigene HIM-Aufnahme des Vero-SARS-CoV-2-Systems, in der zwischen den Mikrovilli der Verozellen die Virenknospen erkennbar sind. Für das Experiment ließen wir Vero-Zellen auf einem Deckgläschen wachsen und infizierten sie mit SARS-CoV-2. Die infizierten Zellen fixierten wir mit 4%iger Formalin- und 1%iger Osmiumtetroxid-Lösung, entwässerten in einer Ethanolreihe und trockneten mittels Kritischpunkttrocknung. Metallisierung oder Kohlebedampfung der Oberfläche waren durch die Verwendung der Ladungskompensation nicht nötig, sodass Artefakte durch diesen Präparationsschritt ausgeschlossen werden können.

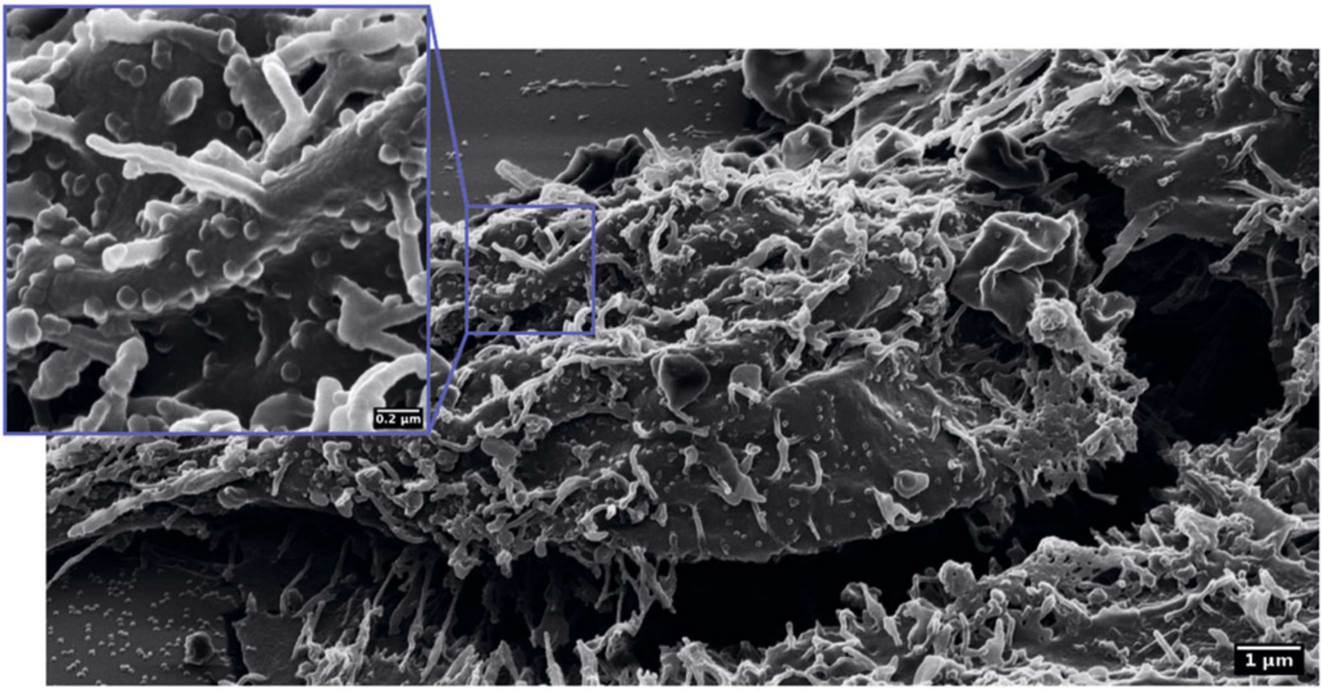

▲ **Abb. 3:** Heliumionenmikrografie einer mit SARS-CoV-2 infizierten Verozelle. In der Ausschnittvergrößerung sind deutlich Knospen an der Zellmembran erkennbar, aus denen neue Virenpartikel hervorgehen. Probe: Institut für Virologie, Universitätsklinikum Leipzig.

## Einblicke in die Ultrastruktur von Mikroben

Wir zeigen die Möglichkeiten der nanoskaligen Strukturaufklärung mikrobieller Proben mittels HIM auf, wobei diese durch das Tripel aus hoher Lateral- und Tiefenauflösung, großer Tiefenschärfe und Ladungskompensation für zahlreiche Aufgaben der REM überlegen ist. Ebenso wichtig wie Strukturaufklärung ist für biologische Fragestellungen jedoch auch die chemische und/oder Isotopen-Nanoanalytik. Die REM hat dabei einen Vorteil gegenüber der HIM: Der Elektronenstrahl erzeugt Röntgenstrahlen, die zur Elementanalyse genutzt werden können. Im Gegensatz dazu entstehen im HIM jedoch keine Röntgenstrahlen. Allerdings lassen sich die in der Probe generierten Sekundärionen detektieren und analysieren.

Hier liegt ein enormes Potenzial, da sich damit – ausreichende Massenauflösung der Spektrometer vorausgesetzt – nicht nur Analysen der chemischen, sondern auch der isotopischen Zusammensetzung der Probe durchführen lassen. So wurden bereits HIM-basierte Sekundärionenmassenspektrometer (HIM-SIMS) mit lateraler Auflösung von ungefähr zehn Nanometer entwickelt und zur Untersuchung der Wechselwirkung von *Daphnia magna* mit $TiO_2$-Nanopartikeln genutzt [13].

Neben den vielfältigen möglichen Anwendungen von HIM-SIMS zur Untersuchung von Wechselwirkungen mineralischer Nanopartikel mit Zellen bzw. Gewebe, ist weiter zu erwarten, dass HIM-SIMS sich hervorragend eignet zur Untersuchung von biologischen Mineralisationsprozessen, etwa der Bildung von Zahn- oder Knochengewebe, der bakteriellen Fällung von Schwermetallverbindungen aus Lösungen oder der mikrobiellen Korrosion. Vielversprechend erscheint auch die bisher nur wenig genutzte Möglichkeit, den Ionenstrahl des HIM als Skalpell mit nanometergenauer Präzision zum Schneiden biologischer Proben zu nutzen, um deren innere Struktur aufzuklären (publizierte Beispiele an Nematoden, Bakterien und Bakteriophagen in [6, 10, 11]).

Auch lassen sich mittels Ionenstrahlschneiden und darauffolgender Mikroskopie nanoskalige Interaktionen zwischen Mikroben und Oberflächen untersuchen [14]. In naher Zukunft könnten durch die Verwendung von Ionendetektoren in Transmissionsgeometrie (z. B. [13]) HIM-Untersuchungen an Dünnschnittproben in Analogie zu TEM möglich werden, was spannende neue Einblicke in die Ultrastruktur von Mikroben erwarten lässt.

War HIM in der Mikrobiologie bisher eher eine Nischentechnik, so lassen ihre vielfältigen Einsatzgebiete als hochauflösende Mikroskopie neue analytische und Ionenstrahl-lithografische Möglichkeiten sowie eine Einbindung in Kryo-Workflows in den kommenden Jahren zahlreiche neue mikrobiologische Anwendungen dieser Technik erwarten.

## Danksagung lung (EFRE) und der Helmholtz Gemeinschaft. ■


Funding note: Open Access funding enabled and organized by Projekt DEAL.

Matthias Schmidt 2001–2006 Physikstudium an der Universität Leipzig mit Schwerpunkt Festkörperphysik. 2007–2012 Doktorand an Universität Leipzig und Helmholtz-Zentrum Dresden-Rossendorf, Promotion bei Prof. Dr. M. Grundmann, Universität Uni Leipzig. 2012–2013 Postdoc, University of Pretoria, Südafrika. Seit 2014 Leiter des Labors für Hochauflösende Mikroskopie am ProVIS Zentrum für Chemische Mikroskopie des Helmholtz-Zentrums für Umweltforschung GmbH — UFZ.

Danksagung

Mein herzlicher Dank gilt James Byrne (University of Bristol, UK) für die Probe der nitratreduzierenden, eisen(II)oxidierenden Bakterienkultur und Jairo H. Moreno Osorio (damals Helmholtz-Zentrum für Umweltforschung — UFZ, Leipzig) für den Chlorella-Biofilm. Nedal Said und Antonis Chatzinotas (Helmholtz-Zentrum für Umweltforschung — UFZ, Leipzig) danke ich sehr für die hervorragende Zusammenarbeit zum Lebenszyklus von Bdellovibrio. Ich danke Uwe Gerd Liebert, Melanie Maier und Grit Szczepankiewicz (Universitätsklinikum Leipzig, Institut für Virologie) herzlich für die mit SARS-CoV-2 infizierten Verozellen.

Des Weiteren danke ich meinen Kolleg:innen vom ProVIS — Zentrum für Chemische Mikroskopie für die hervorragende Zusammenarbeit und die Nutzung des Heliumionenmikroskops. Für die finanzielle Unterstützung von ProVIS danke ich dem Europäischen Fonds für Regionale Entwicklung (EFRE) und der Helmholtz Gemeinschaft.


Literatur

[1] Krause F Das magnetische Elektronenmikroskop und seine Anwendungen in der Biologie Naturwiss 1937 25 817 10.1007/BF01734636
[2] von Borries B Ruska E Ruska H Übermikroskopische Bakterienaufnahmen Wiss Veröff Siemens 1938 17 107 111
[3] Ruska H von Borries B Ruska E Die Bedeutung der Übermikroskopie für die Virusforschung Arch ges Virusforschung 1939 1 155 169 10.1007/BF01243399
[4] Gray T Stereoscan electron microscopy of soil microorganisms Science 1967 155 1668 1670 10.1126/science.155.3770.1668 6020285
[5] Ward B Notte J Economou N Helium ion microscope: a new tool for nanoscale microscopy and metrology J Vacuum Sci Technol B 2006 24 2871 2874 10.1116/1.2357967
[6] Joens MS Huynh C Kasuboski JM Helium ion microscopy (HIM) for the imaging of biological samples at sub-nanometer resolution Sci Rep 2013 3 3514 10.1038/srep03514 24343236 PMC3865489
[7] Moreno-Osorio JH Benettoni P Schmidt M Investigation of architecture development and phosphate distribution in Chlorella biofilm by complementary microscopy techniques FEMS Microbiol Ecol 2019 95 fiz029 30848779 10.1093/femsec/fiz029
[8] Schmidt M Byrne JM Maasilta IJ Bio-imaging with the helium-ion microscope: a review Beilstein J Nanotechnol 2021 12 1 3 10.3762/bjnano.12.1 33489663 PMC7801799
[9] Byrne JM Schmidt M Gauger T Imaging organic-mineral aggregates formed by Fe(II)-oxidizing bacteria using helium ion microscopy Environ Sci Technol Lett 2018 5 209 213 10.1021/acs.estlett.8b00077
[10] — M. Leppänen, L.-R. Sundberg, E. Laanto, G. M. de Freias Almeida, P. Papponen, I.J. Maasilta: Imaging Bacterial Colonies and Phage-Bacterium Interactions at Sub-Nanometer Resolution Using Helium-Ion Microscopy, Adv. Biosyst. 1, 1700070 (2017) DOI: 10.1002/adbi.20170007010.1002/adbi.201700070 32646179
[11] Said N Chatzinotas A Schmidt M Have an ion on it: the life-cycle of Bdellovibrio bacteriovorus viewed by helium-ion microscopy Adv Biosyst 2019 3 1800250 10.1002/adbi.201800250 32627346
[12] Frese N Schmerer P Wortmann M Imaging of SARS-CoV-2 infected Vero E6 cells by helium ion microscopy Beilstein J Nanotechnol 2021 12 172 179 10.3762/bjnano.12.13 33614383 PMC7871036
[13] De Castro O Biesemeier A Serralta E npSCOPE: a new multimodal instrument for in situ correlative analysis of nanoparticles Anal Chem 2021 93 14417 14424 10.1021/acs.analchem.1c02337 34670088
[14] Bandara CD Ballerin G Leppänen M Resolving bio-nano interactions of E.coli bacteria-dragonfly wing interface with helium ion and 3D-structured illumination microscopy to understand bacterial death on nanotopography ACS Biomater Sci Eng 2020 6 3925 3932 10.1021/acsbiomaterials.9b01973 33463326
